# High positive end expiratory pressure levels affect hemodynamics in elderly patients with hypertension admitted to the intensive care unit: a prospective cohort study

**DOI:** 10.1186/s12890-019-0965-9

**Published:** 2019-11-27

**Authors:** Lili Zhou, Guoen Cai, Zhihui Xu, Qinyong Weng, Qinyong Ye, Cunrong Chen

**Affiliations:** 1 Department of Geriatrics, Union Hospital, Fujian Medical University, Fuzhou, Fujian 350001 People’s Republic of China; 2 Department of Critical Care Medicine, Union Hospital, Fujian Medical University, Fuzhou, Fujian 350001 People’s Republic of China; 3 Department of Neurology, Union Hospital, Fujian Medical University, Fuzhou, Fujian 350001 People’s Republic of China

**Keywords:** Positive end expiratory pressure, Hemodynamics, Elderly patients with hypertension, Mechanical ventilation

## Abstract

**Background:**

To study the effects of different positive end expiratory pressure (PEEP) on blood pressure and heart function in elderly patients with hypertension.

**Methods:**

Forty elderly patients above 65 years of age treated with mechanical ventilation were divided into two groups: a control group of non-hypertensive subjects (*n* = 18) and a hypertension group (*n* = 22) patients with essential hypertension. Changes in blood pressure, central venous pressure (CVP), central venous oxygen saturation (ScvO_2_), heart rate, and airway pressure were determined in response to different selected PEEP levels of 0, 2, 4, 6, 8, 10 and 12 cm H_2_O under SIMV(PC) + PSV mode throughout the study.

**Results:**

In both groups, the increase in PEEP led to an increase in CVP and airway pressure. When PEEP was above 4 cm H_2_O in the hypertension group, a decrease in blood pressure and ScvO_2_, and an increase of heart rate were observed. These results indicated that cardiac output significantly decreased.

**Conclusion:**

High levels of PEEP can significantly influence changes in blood pressure and heart function in elderly patients with hypertension.

**Trial registration:**

This trial was retrospectively registered, The Chinese trial registration number is ChiCTR-ROC-17012873. The date of registration is 10-2-2017.

## Background

The number of elderly patients with essential hypertension is increasing annually at a rapid rate worldwide [1, 2]. Recent data from the National Health and Nutrition Examination Survey indicate that 70% of older adults have hypertension [3]. Elastic arteries of elderly hypertensives dilate and stiffen, which decreases arterial capacitance and limited recoil and is thus unable to accommodate the changes that occur during the cardiac cycle [4, 5]. Therefore, if cardiac output decreases, the blood pressure drops immediately in elderly hypertensives.

Multiple methods exist for the measurement of cardiac output. However, accurate measurement of cardiac output, such as that obtained using a SwanGanz catheter and cardiac color ultrasound requires advanced instrumentation. Another important factor for hemodynamic stability is the balance between oxygen delivery (DO_2_) and consumption (VO_2_). The most often used bedside parameter to assess the relationship between oxygen supply and consumption is central venous oxygen saturation (ScvO_2_) [6, 7]. ScvO_2_ decreases under low oxygen supply conditions as in occurs during low cardiac output, decreased hemoglobin, decreased arterial oxygen saturation, or in cases of increased oxygen demand that occurs with shivering, fever, agitation, and hypermetabolic state [8, 9]. Therefore, if hemoglobin, arterial oxygen saturation and oxygen demand are relatively invariant, ScvO_2_ is associated with cardiac output.

Elderly hypertensive patients have poor cardiorespiratory function and often require ventilator assisted breathing [10]. As far as we know, the elasticity of arterial vessels in elderly patients with hypertension is diminished while the adjustment function of arterial vessels is poor; therefore, hemodynamics is susceptible to external factors such as positive pressure ventilation [11]. If positive end expiratory pressure (PEEP) is improperly used, it may have negative effects on blood dynamics and oxygen metabolism in elderly hypertensive patients, and may even cause multiple organ dysfunction and death [12].

This study aimed to explore the effects of different PEEP levels on blood pressure and heart function in elderly patients with hypertension, and to minimize the adverse effects of PEEP on hemodynamics.

## Methods

### Study design

For all selected patients, the SIMV (PC) + PSV ventilator mode (Vela ventilator, American) was utilized to assist breathing. The tidal volume was controlled (10 ml/kg) to maintain pulmonary oxygenation and promote carbon dioxide exhalation, as well as the inhalation time (1.2 s), and respiratory frequency (18 times/min). Oxygen concentration maintenance at 40% made the peripheral oxygen saturation more than 95%. All patients underwent radial artery puncture and subclavian venous catheter. Phlegm was removed from the airway, and subjects were placed at a comfortable 30° half supine position with infusion speed regulated at 40–50 drops/min. Midazolam 0.05 mg/kg/h was used to limit spontaneous breathing. Tidal volume was maintained relatively constant, and changes in blood pressure, central venous pressure (CVP), ScvO_2_, heart rate (HR), and airway pressure were determined in response to different selected PEEP levels of 0, 2, 4, 6, 8, 10 and 12 cm H_2_O. Each PEEP level exposure lasted 2 h. The researchers were blinded to patient treatment conditions. If a significant decrease in blood pressure and an obvious decrease of peripheral oxygen saturation (SpO_2_) were noted, the study was terminated to ensure patient safety. No patients withdrew from the study.

### Participants

**Fig. 1:**
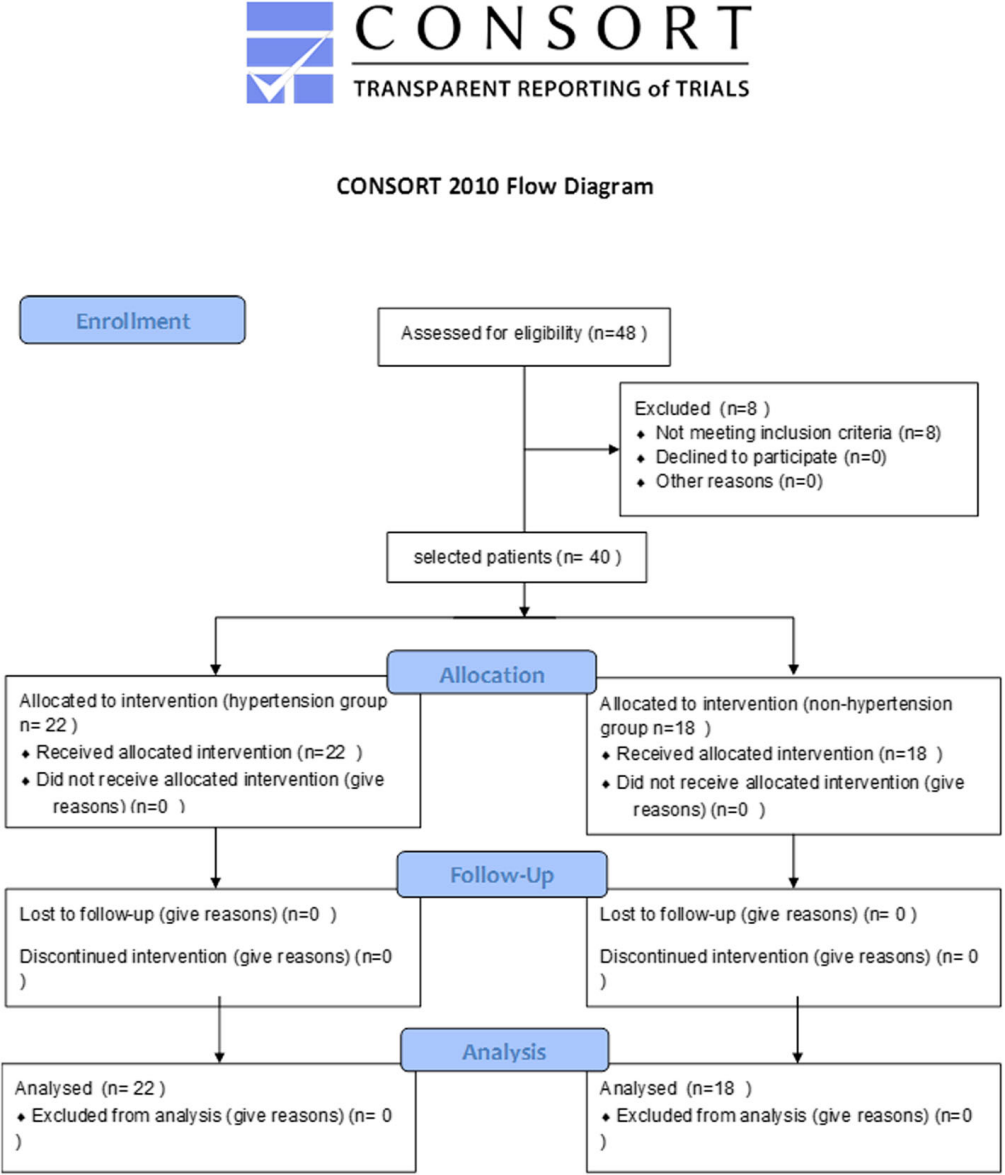
Consort Flow Diagram

This prospective, observational study was conducted between January 2015 and January 2017 at the Intensive Care Unit in Fujian Medical University Union Hospital. Chinese clinical trial registration number is ChiCTR-ROC-17012873. Forty elderly patients over 65 years of age with respiratory failure undergoing mechanical ventilation treatment were divided into two groups: a control group of non-hypertension subjects (*n* = 18) and a hypertension group of patients (*n* = 22) with essential hypertension (Fig. 1). Respiratory failure in these 40 elderly patients resulted from pneumonia. All patients or their legally authorized representatives provided written informed consent. The study was approved by the Ethics Committee of Union Hospital Affiliated to Fujian Medical University. We excluded patients with secondary hypertension, shock, arrhythmia, and severe heart failure (NYHA cardiac function grade III - IV).

### Lung function and hemodynamic measurements

To ensure that the subclavian vein catheter (Arrow, American) was located in the superior vena cava, the position of the catheter was confirmed by bedside X-ray or CT. The subclavian vein catheter was connected to a multi-parameter cardioelectric monitor (GEcicpro/8000i, American) to measure CVP. Extraction of 1.5 ml central venous catheter blood was used by blood gas analysis (i-stat300 blood gas analyzer, Abbott, United States) for ScvO_2_ monitoring. In addition, a multi-parameter cardioelectric monitor continuously assessed radial blood pressure, HR, and SpO_2_. A ventilator continuously monitored peak airway pressure (Ppeak), mean airway pressure (Pmean), tidal volume (VT) and minute ventilation volume (MV).

### Evaluation criteria

Hypertension was defined as systolic blood pressure (SBP) ≥140 mmHg and/or diastolic blood pressure (DBP) ≥90 mmHg under sufficient sedation during mechanical ventilation without man-machine confrontation and/or the self-reported use of antihypertensive medication in the previous 2 weeks [13].

### Statistics analysis

We determined the sample size for the study using statistical analysis software, NCSS PASS. All data were presented as mean ± SD or median (interquartile range) as indicated by data distribution tested by the Shapiro-Wilk test. To compare data between groups, a *t* test, analysis of variance (ANOVA), Mann–Whitney test, or Kruskal–Wallis test was utilized depending on their distribution and number of variables. Correlation was analyzed by Pearson correlation analysis. Confounding factors were controlled by multiple linear regression analysis. Statistical significance was considered at *P* < 0.05.

## Results

### Participants characteristics

Descriptive characteristics are summarized in Table 1. SBP, DBP, mean arterial blood pressure (MABP), serum creatinine (Scr) and uric acid (UA) levels of the hypertension group were elevated compared to the control group (*P* < 0.05). There were no statistically significant differences in age, triglyceride (TG), total cholesterol (TC), low density lipoprotein cholesterin (LDL-C), fasting plasma glucose (FPG), hemoglobin (HB), or body temperature (T) between the two groups (*P* > 0.05). When PEEP was 0 cm H_2_O, there was also no statistically significant difference in CVP between the groups (*P* > 0.05).

**Table 1:** Baseline characteristics. Data are shown as mean ± SD or median (interquartile)

	Control group (*n* = 18)	Hypertension group (*n* = 22)
Sex M/F (n)	12/6	14/8
Age (years)	78.1 ± 9.2	74.5 ± 6.3
SBP (mmHg)	119.1 ± 9.9	151.6 ± 13.2*
DBP (mmHg)	67.4 ± 5.3	82.0 ± 12.0*
MABP(mmHg)	84.7 ± 3.7	105.1 ± 10.1*
Scr (umol/L)	68.0 (48.5,83.3)	110.4 (57.3202.3)*
UA (umol/L)	163.0 (81.5,311.3)	434.5 (312.0,509.0)*
TG (mmol/L)	1.1 (0.8,1.7)	1.7 (1.0,3.0)
TC (mmol/L)	3.6 ± 1.2	3.8 ± 0.9
LDL-C (mmol/L)	2.2 ± 1.0	2.2 ± 0.8
FPG (mmol/L)	7.6 (5.4,8.7)	6.5 (4.8,9.1)
HB (g/L)	102.1 ± 26.0	91.8 ± 21.6
T (°C)	37.1 ± 0.4	36.9 ± 0.5
CVP(mmHg, PEEP 0 cm H_2_O)	7.2 ± 1.2	8.1 ± 3.3

*SBP* Systolic blood pressure, *DBP* Diastolic blood pressure, *MABP* Mean arterial blood pressure, *Scr* Serum creatinine, *UA* Uric acid, *TG* Triglyceride, *TC* Total cholesterol, *LDL-C* Low density lipoprotein cholesterin, *FPG* Fasting plasma glucose, *HB* Hemoglobin, *T* Body temperature, *CVP* Central venous pressure, *PEEP* Positive end expiratory pressure

*: *P*<0.05

### Results per study group

#### Effect of different PEEP levels on blood pressure

**Fig. 2:**
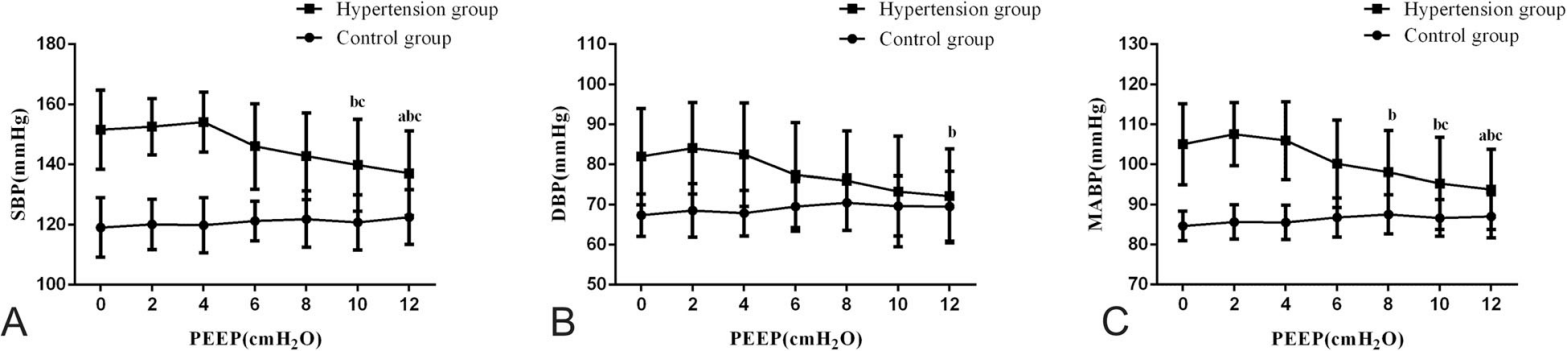
Effect of different PEEP levels on systolic blood pressure (SBP), diastolic blood pressure (DBP), and mean arterial blood pressure (MABP). **a**: Effect of different PEEP levels on SBP, **b**: Effect of different PEEP levels on DBP, **c**: Effect of different PEEP levels on MABP. Data are shown as mean and standard deviation. In the same group of blood pressure, ^a^indicated significant difference (*p* < 0.05) compared with PEEP 0 cmH_2_O. ^b^indicated significant difference compared with PEEP 2 cmH_2_O. ^c^indicated significant difference compared with PEEP 4 cmH_2_O

In the control group, SBP, DBP, and MABP were unaffected by PEEP (*P* > 0.05). In the hypertension group, when PEEP was below 4 cm H_2_O, blood pressure was also unaffected by PEEP (*P* > 0.05). However, when PEEP was above 4 cm H_2_O, this increase in PEEP led to an decrease in blood pressure (*P* < 0.05) (Table 2, Fig. 2). Moreover, in the hypertension group, when PEEP above 4 cm H_2_O, PEEP was negatively correlated with SBP (*r* = − 0.390, *P* < 0.001), DBP (*r* = − 0.266, *P* = 0.005), and MABP (*r* = − 0.374, *P* < 0.001). Using multiple linear regression analysis to control confounding factors, the results showed that with the increase of controlling factors, the negative correlation between PEEP and blood pressure still existed (Table 3).

**Table 2:** Effect of different PEEP levels on hemodynamics. Data are shown as mean ± SD

		PEEP(cmH_2_O)						
		0	2	4	6	8	10	12
SBP (mmHg)	0	119.1 ± 9.9	120.2 ± 8.4	119.9 ± 9.2	121.3 ± 6.6	121.9 ± 9.4	120.8 ± 9.2	122.6 ± 9.1
	1	151.6 ± 13.2	152.6 ± 9.4	154.2 ± 10.0	146.1 ± 14.2	142.8 ± 14.4	139.8 ± 15.3^bc^	137.1 ± 14.1^abc^
DBP (mmHg)	0	67.4 ± 5.3	68.6 ± 6.7	67.9 ± 5.7	69.6 ± 6.2	70.5 ± 6.9	69.7 ± 7.5	69.6 ± 8.7
	1	82.0 ± 12.0	84.1 ± 11.4	82.5 ± 12.9	77.4 ± 13.1	76.0 ± 12.4	73.3 ± 13.8	72.2 ± 11.7^b^
MABP (mmHg)	0	84.7 ± 3.7	85.7 ± 4.3	85.6 ± 4.3	86.8 ± 4.9	87.6 ± 4.9	86.7 ± 4.6	87.1 ± 5.4
	1	105.1 ± 10.1	107.6 ± 7.9	106.0 ± 9.7	100.2 ± 10.9	98.1 ± 10.4^b^	95.3 ± 11.5^bc^	93.8 ± 10.0^abc^
CVP (mmHg)	0	7.2 ± 1.2	7.4 ± 1.2	7.6 ± 1.2	7.8 ± 1.3	8.0 ± 1.2	8.2 ± 1.3	8.4 ± 1.3
	1	8.1 ± 3.3	8.4 ± 3.1	9.0 ± 4.2	8.7 ± 2.6	9.0 ± 2.9	9.1 ± 2.8	9.3 ± 2.9
ScvO_2_ (%)	0	68.4 ± 7.3	68.6 ± 5.8	69.3 ± 6.0	69.9 ± 7.4	71.3 ± 5.7	70.4 ± 8.0	72.0 ± 6.6
	1	67.3 ± 8.5	69.3 ± 8.9	70.8 ± 7.6	67.8 ± 5.7	65.3 ± 6.4	64.0 ± 5.7^c^	61.9 ± 6.2^cd^
HR (bpm)	0	82.6 ± 12.5	82.9 ± 12.7	83.3 ± 12.9	83.8 ± 13.5	82.1 ± 13.2	82.6 ± 13.3	82.7 ± 12.8
	1	80.2 ± 12.5	78.5 ± 12.2	76.9 ± 10.4	80.9 ± 12.7	82.7 ± 10.7	85.1 ± 9.8	88.2 ± 11.5^c^
Ppeak (cmH_2_O)	0	20.4 ± 4.9	21.5 ± 4.6	22.3 ± 4.4	23.6 ± 4.5	24.6 ± 4.4	25.7 ± 4.5^a^	27.3 ± 4.8^ab^
	1	20.8 ± 4.1	21.8 ± 4.3	23.3 ± 4.6	24.6 ± 5.1	26.1 ± 4.9^a^	27.4 ± 4.8^ab^	29.3 ± 4.7^abc^
Pmean (cmH_2_O)	0	10.1 ± 2.1	11.1 ± 2.2	11.9 ± 2.3	13.1 ± 2.5^a^	13.9 ± 2.2^ab^	15.1 ± 2.4^abc^	16.2 ± 2.7^abcd^
	1	10.3 ± 2.3	11.2 ± 2.0	12.3 ± 2.1	13.7 ± 2.2^ab^	14.7 ± 2.1^abc^	16.0 ± 2.2^abcd^	17.5 ± 2.2^abcde^
VT (ml)	0	644.6 ± 95.3	649.7 ± 96.8	654.0 ± 96.1	651.9 ± 94.3	642.6 ± 106.2	646.3 ± 106.4	645.3 ± 107.2
	1	584.0 ± 119.1	587.0 ± 118.3	591.1 ± 114.1	589.6 ± 115.4	586.9 ± 117.7	587.4 ± 116.3	585.3 ± 117.3
MV (L/min)	0	11.7 ± 1.6	11.7 ± 1.6	11.8 ± 1.6	11.8 ± 1.5	11.6 ± 1.8	11.7 ± 1.8	11.7 ± 1.9
	1	10.5 ± 2.1	10.6 ± 2.1	10.8 ± 2.0	10.7 ± 2.0	10.8 ± 2.1	10.9 ± 2.0	10.7 ± 2.0
SpO_2_	0	100.0 ± 0.0	100.0 ± 0.0	100.0 ± 0.0	100.0 ± 0.0	100.0 ± 0.0	100.0 ± 0.0	100.0 ± 0.0
(%)	1	99.9 ± 0.5	99.9 ± 0.3	100.0 ± 0.2	100.0 ± 0.2	100.0 ± 0.2	100.0 ± 0.2	99.9 ± 0.5

*SBP* Systolic blood pressure, *DBP* Diastolic blood pressure, *MABP* Mean arterial blood pressure, *CVP* Central venous pressure, *ScvO*_*2*_ Central venous oxygen saturation, *HR* Heart rate, *Ppeak* Peak airway pressure, *Pmean* Mean airway pressure, *VT* Tidal volume, *MV* Minute ventilation volume, *SpO*_*2*_ Peripheral oxygen saturation; 0: Control group; 1: Hypertension group;

^a^*P* < 0.05, compared with PEEP 2 cm H_2_O: ^b^*P* < 0.05, compared with PEEP 4 cm H_2_O: ^c^*P* < 0.05, compared with PEEP 6 cm H_2_O: ^d^*P* < 0.05, compared with PEEP 8 cm H_2_O: ^e^*P* < 0.05. In the same group of blood pressure, compared with PEEP 0 cm H_2_O: ^a^*P* < 0.05, compared with PEEP 2 cm H_2_O: ^b^*P* < 0.05, compared with PEEP 4 cm H_2_O: ^c^*P* < 0.05, compared with PEEP 6 cm H_2_O: ^d^*P* < 0.05, compared with PEEP 8 cm H_2_O: ^e^*P* < 0.05

**Table 3:** Multiple linear regression analysis of blood pressure and PEEP

Hypertension group (PEEP≥4cmH_2_O)	Model1	Model 2	Model 3
SBP			
*Beta*	−0.417	− 0.428	−0.473
*P*	< 0.001	< 0.001	< 0.001
DBP			
*Beta*	−0.277	−0.307	− 0.341
*P*	0.004	0.002	0.001
MABP			
*Beta*	−0.394	− 0.423	−0.467
*P*	< 0.001	< 0.001	< 0.001

Model 1: Adjustment for age and gender; Model 2: Adjustment for age, gender, triglyceride, total cholesterol, low density lipoprotein cholesterin, fasting plasma glucose, serum creatinine and uric acid; Model 3: Adjustment for hemoglobin, body temperature, minute ventilation volume and peripheral oxygen saturation on the basis of Model 2

*SBP* Systolic blood pressure, *DBP* Diastolic blood pressure, *MABP* Mean arterial blood pressure

#### Effect of different PEEP levels on cardiac function

**Fig. 3:**
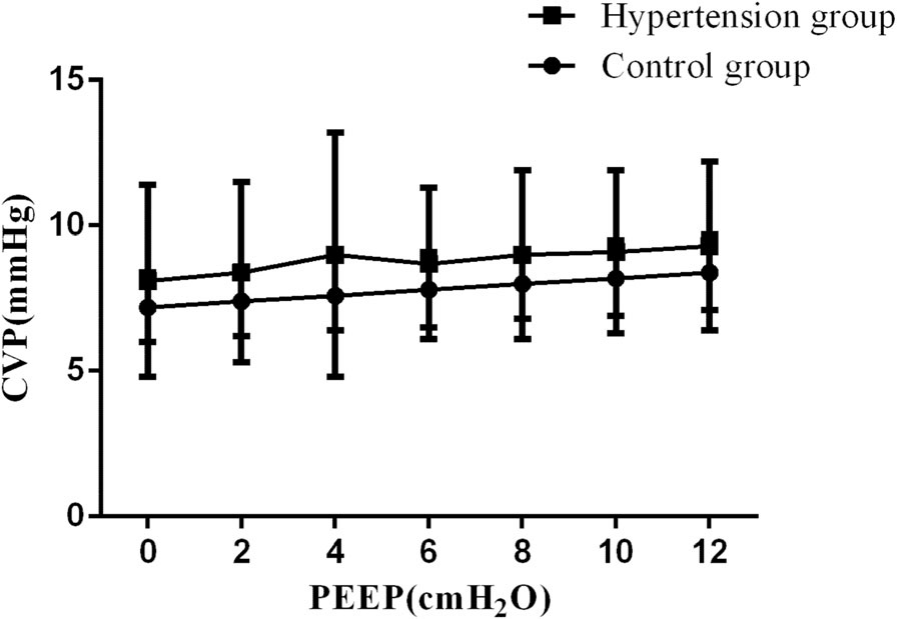
Effect of different PEEP levels on central venous pressure (CVP). Data are shown as mean and standard deviation

**Fig. 4:**
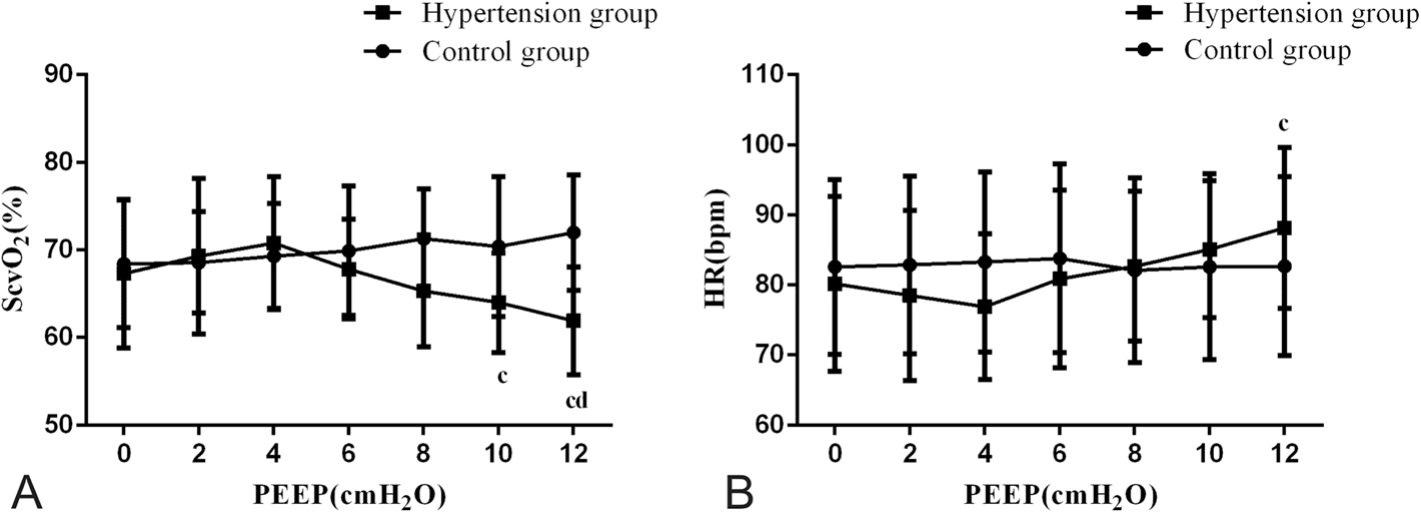
4 Effect of different PEEP levels on central venous oxygen saturation (ScvO_2_) and heart rate (HR). **a**: Effect of different PEEP levels on ScvO_2_, **b**: Effect of different PEEP levels on HR. Data are shown as mean and standard deviation. In the same group of blood pressure, ^c^indicated significant difference (*p* < 0.05) compared with PEEP 4 cmH_2_O. ^d^indicated significant difference compared with PEEP 6 cmH_2_O

In both groups, an increase in PEEP led to an increase of CVP (Table 2, Fig. 3). Moreover, PEEP was positively correlated with the CVP (*r* = 0.149, *P* = 0.013). With an increase in controlling factors, a positive correlation between PEEP and CVP was still observed (Table 4). In the control group, HR and ScvO_2_ were unaffected by PEEP (*P* > 0.05). In the hypertension group, when PEEP was less than 4 cm H_2_O, HR and ScvO_2_ were also unaffected (*P* > 0.05). However, when PEEP was above 4 cm H_2_O, an increase of HR and a decrease of ScvO_2_ were observed (Table 2, Fig. 4). Furthermore, in the hypertension group, when PEEP was above 4 cm H_2_O, PEEP was positively correlated with HR (*r* = 0.333, *P* < 0.001) and negatively correlated with ScvO_2_ (*r* = − 0.441, *P* < 0.001). Using multiple linear regression analysis to control confounding factors, the correlation between PEEP and HR, ScvO_2_ still existed (Table 5).

**Table 4:** Multiple linear regression analysis of CVP and PEEP

All subjects	Model1	Model2	Model3
CVP			
*Beta*	0.150	0.152	0.174
*P*	0.012	0.012	0.004

Model 1: Adjustment for age and gender; Model 2: Adjustment for age, gender, triglyceride, total cholesterol, low density lipoprotein cholesterin, fasting plasma glucose, serum creatinine and uric acid; Model 3: Adjustment for hemoglobin, body temperature, minute ventilation volume and peripheral oxygen saturation on the basis of Model 2

*CVP* Central venous pressure

**Table 5:** Multiple linear regression analysis of cardiac function and PEEP

Hypertension group (PEEP ≥4 cm H_2_O)	Model 1	Model 2	Model 3
ScvO_2_			
*Beta*	−0.455	− 0.552	−0.592
*P*	< 0.001	< 0.001	< 0.001
HR			
*Beta*	0.333	0.345	0.428
*P*	< 0.001	< 0.001	< 0.001

Model 1: Adjustment for age and gender; Model 2: Adjustment for age, gender, triglyceride, total cholesterol, low density lipoprotein cholesterin, fasting plasma glucose, serum creatinine and uric acid; Model 3: Adjustment for hemoglobin, body temperature, minute ventilation volume and peripheral oxygen saturation on the basis of Model 2

*ScvO*_*2*_ Central venous oxygen saturation, *HR* Heart rate

#### Effects of different PEEP levels on airway pressure

**Fig. 5:**
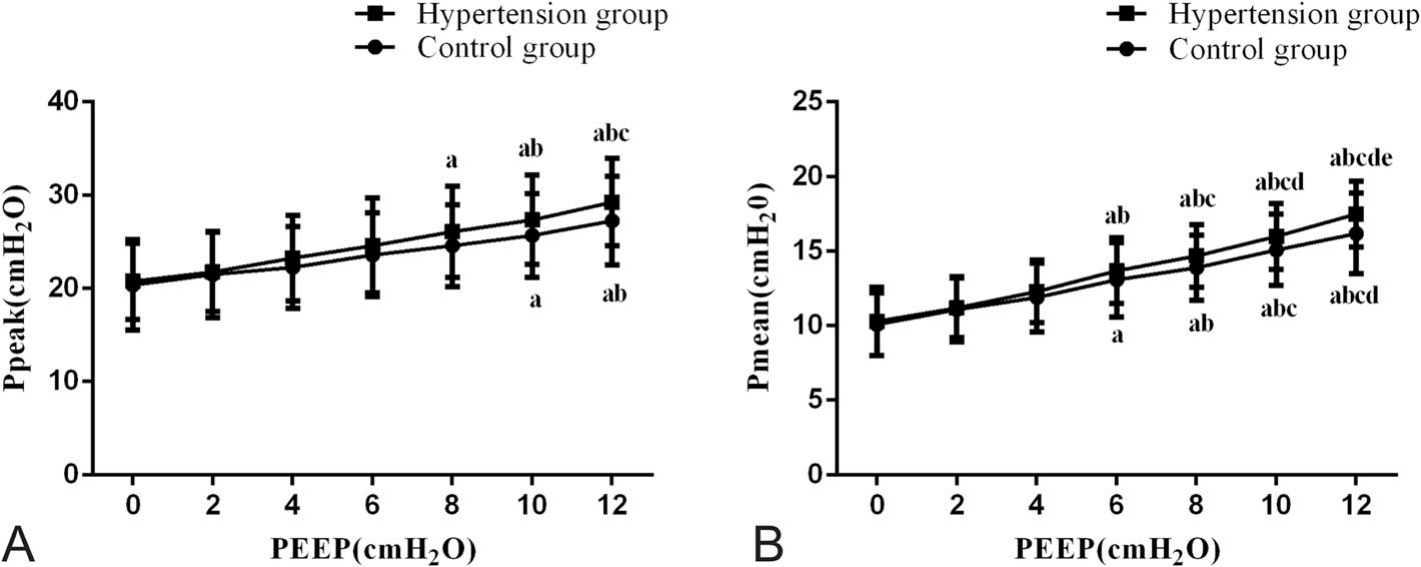
Effect of different PEEP levels on peak airway pressure (Ppeak) and mean airway pressure (Pmean). **a**: Effect of different PEEP levels on Ppeak, **b**: Effect of different PEEP levels on Pmean. Data are shown as mean and standard deviation. In the same group of blood pressure, ^a^indicated significant difference (*p* < 0.05) compared with PEEP 0 cmH_2_O. ^b^indicated significant difference compared with PEEP 2 cmH_2_O. ^c^indicated significant difference compared with PEEP 4 cmH_2_O. ^d^indicated significant difference compared with PEEP 6 cmH_2_O. ^e^indicated significant difference compared with PEEP 8 cmH_2_O

In both groups, VT, MV, and SpO_2_ were unaffected by PEEP, but the increase in PEEP led to an increase of Ppeak and Pmean (Table 2, Fig. 5). In addition, PEEP was positively correlated with the Ppeak (*r* = 0.489, *P* < 0.001) and Pmean (*r* = 0.708, *P* < 0.001). Using multiple linear regression analysis to control confounding factors, a correlation between PEEP and airway pressure was still observed (Table 6).

**Table 6:** Multiple linear regression analysis of airway pressure and PEEP

All subjects	Model1	Model2	Model3
Ppeak			
*Beta*	0.489	0.514	0.515
*P*	< 0.001	< 0.001	< 0.001
Pmean			
*Beta*	0.713	0.740	0.744
*P*	< 0.001	< 0.001	< 0.001

Model 1: Adjustment for age and gender; Model 2: Adjustment for age, gender, triglyceride, total cholesterol, low density lipoprotein cholesterin, fasting plasma glucose, serum creatinine and uric acid; Model 3: Adjustment for hemoglobin, body temperature, minute ventilation volume and peripheral oxygen saturation on the basis of Model 2

*Ppeak* Peak airway pressure, *Pmean* Mean airway pressure

## Discussion

PEEP is applied during the end of expiration to maintain alveolar pressure above atmospheric pressure. The benefit of PEEP has been demonstrated in terms of preventing collapsed alveoli and improving oxygenation [14, 15]. However, applying PEEP may affect cardiac function and vital organ perfusion via complex mechanisms, especially for elderly hypertensives [16]. Our study found that when PEEP was below 4 cm H_2_O in the both control and hypertension groups, blood pressure was unaffected by PEEP. However, when PEEP was above 4 cm H_2_O, the increase in PEEP led to decreased blood pressure and PEEP was negatively correlated with blood pressure in the hypertension group. Upon control confounding factors, this negative correlation still existed. This observation may be due to diminished elasticity in elderly hypertensives’ blood vessels and subsequent functional decline, suggesting a high level of PEEP would be a negative influence on hemodynamics [17].

To further study why elevated PEEP induced a drop in blood pressure, we explored the relationship between PEEP and cardiac function. As volume status was of high importance for hemodynamic instability in patients ventilated with high tidal volume and different PEEP levels, a sedative drug called midazolam was used to minimize spontaneous breathing, and tidal volume was maintained relatively constant in our study. Moreover, prior to the study, when PEEP was 0 cm H_2_O, there was no statistically significant difference in CVP between the hypertension and control groups. This result indicated that initial fluid status was similar between the two groups. During our study we controlled patients’ body position and infusion speed to ensure that the two groups’ fluid intake was as similar as possible. We found that in both groups, VT, MV, and SpO_2_ were unaffected by PEEP, but increased PEEP led to an increase of CVP and airway pressure. When PEEP was raised above 4 cm H_2_O, in the hypertension group, the increase in PEEP led to a reduction in ScvO_2_ and an increase in HR. Furthermore, PEEP was positively correlated with HR and negatively correlated with the ScvO_2_. To control for confounding factors such as hemoglobin, arterial oxygen saturation and oxygen demand, we found that the correlation between PEEP and HR and ScvO_2_ still existed. This indicated that elevated levels of PEEP caused decreased cardiac output in elderly hypertensive patients [18].

In general, the increase in PEEP caused higher airway pressure which resulted in increased intrathoracic pressure (ITP) [19]. Elevation of CVP by increasing ITP resulted in a reduction in venous return [20]. However, for non-hypertensive subjects, the effects on increasing ITP on venous return, especially by PEEP, did not always lead to decreased cardiac output because of their strong cardiovascular regulation [21]. In elderly hypertensive patients, the thickened and stiffened vein wall caused slower venous return than that of non-hypertension subjects, which resulted in reduced right ventricular (RV) preload [22]. PEEP not only decreased RV preload by impeding systemic venous return, but also increased RV afterload in combination with high tidal volume. In our study, we used high tidal volume of 10 ml/kg body weight to maintain pulmonary oxygenation and promote carbon dioxide exhalation. High PEEP with high tidal volume led to increased end inspiratory lung volume, which was responsible for the RV afterload. Vieilaard-Baron [23] and Protti [24] demonstrated that afterload for RV did not influence capillary compression, but rather the end inspiratory lung volume that caused this compression and reduced the flow through the lung. As a result, the reduction in RV ejection caused by PEEP and high tidal volume decreased left ventricular filling in both groups [25]. However, the hemodynamics of elderly hypertensive patients was more affected by blood volume deficiency than non- hypertensive patients. Therefore, the lower the stroke volume, the faster HR that occurs for elderly hypertensive individuals [26]. The high tidal volume was able to reduce flow through the lung and decrease left ventricular filling, so it was also used to highlight the difference in the response to hypovolemia between the two groups in the present study.

In our study, we found that the increase in PEEP led to an increase in CVP but a decrease in cardiac output. Studies assessing fluid status have shown that CVP monitoring is as effective, and safer, than more invasive means such as pulmonary artery occlusion pressure [27]. It is clear, however, that CVP does not tell the entire story, as patients with positive pressure ventilation may have high CVP, but reduced circulating volume [28]. Currently, many scholars have not advocated the use of CVP to predict changes in pre-cardiac load in mechanical ventilation [29, 30]. However, accurate measurement of cardiac output, such as by SwanGanz catheter and cardiac color ultrasound requires advanced instrumentation and incurs greater cost [31]. Recent studies have found that ScvO_2_ can be used to evaluate the condition of left cardiac ejection, which is simple and reliable and can be used in clinical application [18]. A study by Lanspa et al. reported that assessment of cardiac function often relied on ScvO_2_, with higher ScvO_2_ corresponding with increased cardiac output [32]. As such, our study used ScvO_2_ to reflect left cardiac ejection.

For elderly hypertensive patients, this study showed that PEEP of approximately 4 cm H_2_O could have maximal impact on improving oxygenation, and minimize its impact on hemodynamics. However, due to the limited patient source, the appropriate PEEP value setting should be confirmed by the large sample multicenter clinical study.

## Conclusions

Traditional static index CVP cannot accurately reflect changes in cardiac preload with positive pressure ventilation, though ScvO_2_ shows obvious advantages, which can be used to predict cardiac preload under mechanical ventilation. For elderly patients with hypertension, low levels of PEEP have less effect on blood pressure and cardiac output, while higher levels of PEEP can significantly affect blood pressure and cardiac output. Therefore, the effects of PEEP on the hemodynamics of elderly patients with hypertension should be taken into consideration.

## Data Availability

The datasets used and/or analysed during the current study are available from the corresponding author on reasonable request.

## Abbreviations

ANOVA: Analysis of variance
CVP: Central venous pressure
DBP: Diastolic blood pressure
DO_2_: Oxygen delivery
FPG: Fasting plasma glucose
HB: Hemoglobin
HR: Heart rate
ITP: Increased intrathoracic pressure
LDL-C: low density lipoprotein cholesterin
MABP: Mean arterial blood pressure
MV: Minute ventilation volume
PEEP: Positive end expiratory pressure
Pmean: Mean airway pressure
Ppeak: Peak airway pressure
PVR: Pulmonary vascular resistance
RV: Right ventricular
SBP: Systolic blood pressure
Scr: Serum creatinine
ScvO_2_: Central venous oxygen saturation
SpO_2_: Peripheral oxygen saturation
T: Body temperature
TC: Total cholesterol
TG: Triglyceride
UA: Uric acid
VO_2_: Consumption
